# Prevalence and Trends of Basic Activities of Daily Living Limitations in Middle-Aged and Older Adults in the United States

**DOI:** 10.3390/epidemiologia4040040

**Published:** 2023-11-09

**Authors:** Halli Heimbuch, Yeong Rhee, Marty Douglas, Kirsten Juhl, Kelly Knoll, Sherri Stastny, Ryan McGrath

**Affiliations:** 1 Healthy Aging North Dakota (HAND), North Dakota State University, Fargo, ND 58102, USA; 2 Department of Health, Nutrition, and Exercise Sciences, Fargo, ND 58108, USA; 3 Sanford Health, Fargo, ND 58102, USA; 4 Department of Internal Medicine, University of North Dakota, Grand Forks, ND 58202, USA; 5 Fargo VA Healthcare System, Fargo, ND 58102, USA; 6 Department of Geriatrics, University of North Dakota, Grand Forks, ND 58202, USA; 7 Alliance for Research in Exercise, Nutrition and Activity (ARENA), University of South Australia, Adelaide 5000, Australia

**Keywords:** epidemiology, geriatrics, population surveillance, self-care

## Abstract

Background: Population-level surveillance of the prevalence and trends of basic self-care limitations will help to identify the magnitude of physical disablement in the rapidly growing older American demographic. We sought to evaluate the prevalence and trends of activities of daily living (ADL) limitations in the United States. Methods: The analytic sample included 30,418 Americans aged ≥50 years from the 2006–2018 waves of the Health and Retirement Study. ADLs were self-reported. Weighted prevalence estimates were presented, and trends analyses were performed. Results: Although overall ADL disability prevalence was 16.5% (95% confidence interval: 15.8–17.2) in 2018, there were no changes in limitations during the study period (*p* = 0.52). Older adults had a greater ADL disability prevalence than middle-aged adults (*p* < 0.001). While older persons experienced a declining trend of ADL limitations (*p* < 0.001), middle-aged persons had an increasing trend (*p* < 0.001). Males had a lower ADL limitation prevalence than females (*p* < 0.001). Hispanic and non-Hispanic Black had a higher ADL disability prevalence than non-Hispanic White (*p* < 0.001). Conclusions: This investigation revealed that while the estimated prevalence of ADL limitations in the United States was substantial, changes in such limitations were not observed. Our findings can help guide ADL screening, target sub-populations with an elevated ADL limitation prevalence, and inform interventions.

## 1. Introduction

The older adult population is rapidly increasing worldwide [1]. In the United States, the older demographic is expected to elevate by approximately 113% by the year 2030 [2]. This growth may especially strain the United States healthcare systems, as many older Americans are living with age-related diseases and disabilities [3,4]. For example, over USD 860 billion has been linked to disability-associated healthcare expenditures, with about 54% of Medicare expenses and 72% of Medicaid expenditures being related to disability [5]. Age-related disabilities can also restrict independence and reduce quality of life [6]. Moreover, receiving some types of care for such disabilities can become financially burdensome, with the average annual cost of private nursing home care in the United States being just under USD 100,000 [7]. As such, disability during aging will remain on the forefront of healthcare for older adults.

Limitations in performing activities of daily living (ADL) are a frequently observed type of age-related disability. ADLs evaluate a person’s ability to complete basic self-care tasks, such as dressing and eating [6]. The presence of ADL disability may occur acutely or chronically, in that a fall may abruptly generate an ADL limitation, or declining physical function may contribute to the loss of abilities over time [8,9]. Accordingly, the burden of ADL disability is substantial, and ADL limitations are associated with early all-cause mortality [10,11,12]. Therefore, routinely examining ADLs is critical for the prevention and treatment of functional limitations. While the presence of an ADL disability is related to adverse health outcomes, ADL limitations can be transient, and recovery from a functional disability is possible [12,13]. Accordingly, surveillance of ADL limitations may help inform referrals of older adults to primary and secondary interventions.

Several investigations have previously evaluated the prevalence and trends of ADL limitations at a population-level and have indicated that there is a considerable prevalence of ADL limitations [14,15,16,17,18]. For example, an investigation suggested a flattening of activity limitations for older adults [15], while other studies have revealed a differential ADL limitation prevalence [14,15,16,17,18]. Continual monitoring of ADL disability prevalence, including limitations in individual tasks and different sociodemographic groups, is important for providing precision to ADL care, informing screening, identifying populations at risk for functional disability, and guiding interventions. While the findings from previous research are indeed valuable, this investigation adds updated information to help meet the demand for continual surveillance of ADLs. We sought to examine the prevalence and trends of ADL limitations among middle-aged and older adults in the United States.

## 2. Materials and Methods

### 2.1. Participants

We performed a secondary analysis of data from the 2006–2018 waves of the RAND Health and Retirement Study (HRS). The 2006 wave was selected to align with the concluding year of ADL disability prevalence estimates from other reports [16], whereas the 2018 wave presented the most recent HRS data available for ADLs. The HRS utilizes a longitudinal-panel design for observing health and economic factors during aging [19]. Persons must be aged at least 50 years to be included in the HRS, and new participant cohorts are added to maintain a nationally representative sample of the United States population [20]. Individuals in the HRS are interviewed biennially and followed up until death. Response rates for the HRS have been routinely >80% [21]

The HRS uses a complex multistage probability design, which includes geographical stratification and oversampling for certain demographic groups. The University of Michigan Health Sciences and Behavioral Sciences Institutional Review Board approved HRS protocols, and participants provided their written informed consent before study entry. Additional details about the HRS are available elsewhere [22].

### 2.2. Measures

Respondents told interviewers their age, sex, race, and ethnicity. Respondents also told interviewers about their ability to perform six ADLs at each wave: walk across a small room, dress, bathe, eat, transfer in-and-out of bed, and use a toilet. Those indicating difficulty or an inability to complete a specific ADL were classified as having a limitation in that individual ADL task [12]. Similarly, persons reporting a difficulty or an inability to execute any ADL task were considered as having an ADL disability [15].

### 2.3. Statistical Analysis

All analyses were conducted with SAS 9.4 software (SAS Institute, Cary, NC, USA). HRS analytic guidelines informed our analyses. Survey weights were used to generate nationally representative prevalence estimates. Descriptive characteristics were shown as unweighted mean ± standard deviation and frequency (percentage) for continuous and categorical variables, respectively, to increase interpretability. Prevalence estimates for persons with limitations in specific ADL tasks were presented at each wave, and the overall prevalence of ADL limitations were similarly presented at each wave. ADL disability prevalence estimates were thereafter sub-grouped by age (50–64 years (middle-aged); ≥65 years (older)), sex (male, female), and race and ethnicity (Hispanic, non-Hispanic Black, non-Hispanic Other, non-Hispanic White). Weighted prevalence estimates were coupled with 95% confidence intervals (CI).

Distinct weighted multilevel logistic regression models analyzed trends in ADL disability for overall, age group, sex, and race and ethnicity. Repeated measures of individual participants in several waves were modelled using a random intercept for each participant to account for the longitudinal design. The dichotomous outcome was ADL disability in each model. For the overall model, the only explanatory variable was time (i.e., survey wave). For examining age group trends, another model adjusted for time, age group (reference: middle-aged), and the time-by-age group interaction. Further, time, sex (reference: female), and the interaction between time and sex were modeled for assessing trends by sex. In the last model, we included time, race and ethnicity (reference group: non-Hispanic White), and the interaction of these explanatory variables. An alpha level of 0.05 was utilized for all analyses.

## 3. Results

The overall unweighted descriptive characteristics of the 30,418 participants are shown in Table 1. Participants were aged 63.4 ± 11.1 years and were mostly female (56.8%). The estimated prevalence of individual ADL limitations is presented in Table 2. Limitations in dressing consistently had the highest prevalence estimates, while limitations in eating had the lowest. For example, in the 2018 wave, the estimated prevalence of having limitations with dressing was 9.4% (CI: 8.8, 9.9), whereas the prevalence of having limitations with eating was 3.4% (CI: 3.1, 3.8). The overall estimated prevalence of ADL disability in the United States is presented in Table 3. Although the estimated prevalence of ADL disability went from 17.8% (CI: 17.2, 18.5) in the 2006 wave to 16.5% (CI: 15.8, 17.2) in the 2018 wave, this downward trend was not statistically significant (*p* = 0.52).

Table 4 shows the prevalence estimates of ADL limitations by age group. Older Americans had a greater estimated prevalence of ADL limitations relative to middle-aged Americans (*p* < 0.001). For example, the estimated prevalence of ADL disability in older Americans was 20.2% (CI: 19.2, 21.2) in the 2018 wave, and the aligning ADL disability prevalence estimate in middle-aged Americans was 12.7% (CI: 11.7, 13.8). ADL disability significantly increased in middle-aged Americans over time (*p* < 0.001), while ADL limitations decreased in older Americans (*p* < 0.001). Table 5 presents the estimated prevalence of ADL disability by sex. Males had a significantly lower estimated prevalence of ADL limitations relative to females (*p* < 0.001). In the 2018 wave, the estimated prevalence of ADL disability in males was 14.4% (CI: 13.4, 15.5), while the prevalence estimates were 18.3% (CI: 17.3, 19.3) in females. However, there were no changes in ADL disability over time for both males (*p* = 0.06) and females (*p* = 0.11).

The estimated prevalence of ADL disability by race and ethnicity is shown in Table 6. Persons identifying as Hispanic (*p* < 0.001) and non-Hispanic Black (*p* < 0.001) had a significantly higher estimated prevalence of ADL limitations compared to non-Hispanic White. For example, in the 2018 wave, the estimated prevalence of ADL disability was 23.2% (CI: 20.8, 25.7) in Hispanic, 24.4% (CI: 22.4, 26.5) in non-Hispanic Black, and 14.3% (CI: 13.5, 15.2) in non-Hispanic White. Supplementary Table S1 shows the results for the ADL trends analyses.

## 4. Discussion

The principal findings of this investigation revealed that, while many people in the United States are living with ADL limitations, significant changes in such limitations from 2006–2018 were not observed. Older Americans had a higher estimated prevalence of ADL limitations compared to middle-aged Americans; although the prevalence of ADL disability in older Americans declined, the prevalence of ADL disability in middle-aged Americans increased. Males had a lower ADL disability prevalence relative to females. Moreover, Hispanic and non-Hispanic Black had a higher prevalence of ADL limitations than non-Hispanic White. Our findings should be used to inform ADL screening, target sub-groups of Americans with an elevated prevalence of ADL limitations, and guide intervention framework.

The substantial prevalence of ADL disability in Americans from 2006–2018 aligns with previous findings of Seeman et al. [16] on ADL disability prevalence in 1988–1994 and 1999–2004. However, our trends in ADL disability differ from those observed by Seeman et al., possibly because of dissimilar population characteristics and study years examined. A greater estimated prevalence of ADL limitations in older Americans relative to middle-aged Americans was consistent with other investigations evaluating ADL disability in other countries [23]. These age-related ADL findings were relatively unsurprising, as physical and cognitive functioning usually decline with age, and this decline is associated with ADLs [1,24].

The decreasing estimated ADL prevalence among older Americans during the study period could be attributed to factors that foster the prevention of ADL loss and the recovery of ADLs [13]. The increasing prevalence of ADL disability among middle-aged Americans during the study period aligned with another study [18]. This could be related to morbidities that lead to disability such as diabetes [25]. Another reason for this observation could be sedentary behavior, especially considering the high prevalence of physical inactivity in the United States [26]. Furthermore, physical inactivity may influence the risk of type 2 diabetes, obesity, cardiovascular disease, and other chronic diseases [26].

Our finding of a higher ADL limitation prevalence in females than in males is consistent with the findings of another investigation assessing functional disability [11]. This observation might be explained by the “male-female health survival paradox”, in which females typically have greater longevity than males but often have poorer health [27]. A greater estimated prevalence of ADL limitations in Hispanic and non-Hispanic Black compared to non-Hispanic White aligned with the observations of Tipirneni et al. [17]. Health disparities related to sociodemographic factors may help to explain these results [28,29]. Continual monitoring and intervention to assist in the maintenance and recovery of ADLs in these sub-groups are warranted.

Some study limitations should be noted. Our analyses were stratified by age, gender, and race and ethnicity; however, other sub-group analyses may be relevant and should be considered for future investigations as appropriate. For example, stratifying prevalence estimates by regions of the United States may help guide the customization of interventions according to geographic location. While we used a conventional ADL limitation definition, the degree of limitation in ADL tasks, and interpersonal and intrapersonal differences in these limitations should be considered in future investigations. First, ADL tasks vary in complexity. Some ADL tasks, such as walking across a small room, may require more general skills, including gross motor and perceptual abilities [6]. Meanwhile, other ADL tasks, such as bathing and dressing, may demand a wider variety of both general and detailed skills, including cognitive, motor, and perceptual abilities. Second, environmental and interpersonal factors may influence ADL performance. Examples of these factors include the coexistence of multiple ADL limitations, the variation in materials and the environment involved in an ADL task, and the extent and type of assistance used for performing an ADL task. For example, using garments with difficult closures, such as buttons, ties, and zippers, may require more dexterity relative to using garments without closures [6]. Moreover, ADLs are of a physical nature and include fundamental skills for basic self-care, while separate tasks such as instrumental activities of daily living (IADL) are necessary for independent living [6]. Accordingly, our findings for ADL limitation prevalence and trends differ from those examining IADLs [30]. Further investigating the extent of difficulty in performing ADLs and factors that can impact ADL ability may help inform interventions for prevention, treatment, and recovery.

## 5. Conclusions

While the estimated prevalence of ADL limitations in the United States is high, there were no observed changes in ADL limitations from 2006–2018 in Americans aged at least 50 years. Greater ADL disability prevalence estimates were observed among older adults compared to middle-aged adults. Although ADL disability prevalence approximations were higher for females, they were lower for males. In addition, prevalence estimations of ADL limitations were higher in non-Hispanic Black and Hispanic than in non-Hispanic White. Monitoring ADLs remains an easy and effective method for assessing age-related disability. Insights into prevalence and trends in ADL limitations among Americans are imperative for informing ADL screening, reaching population sectors with elevated prevalence of ADL disability, and advising interventions.

## Figures and Tables

**Table 1:** Unweighted descriptive characteristics of the participants.

Variable	Overall (*n* = 30,418)
Age (years)	63.4 ± 11.1
Age Category (*n* (%))	
Middle-Aged Adult	17,971 (59.1)
Older Adult	12,447 (40.9)
Sex (*n* (%))	
Male	13,143 (43.2)
Female	17,275 (56.8)
Race and Ethnicity (*n* (%))	
Hispanic	4220 (13.9)
Non-Hispanic Black	5983 (19.7)
Non-Hispanic Other	1238 (4.0)
Non-Hispanic White	18,977 (62.4)

**Table 2:** Overall prevalence estimates of individual basic self-care limitations.

Variables	Weighted Frequency (N)	Weighted Prevalence (%)	95% Confidence Interval
2006 Wave			
Walk Across a Small Room	5,683,145	7.3	6.9, 7.7
Dressing	7,840,499	10.0	9.5, 10.5
Bathe	5,611,356	7.2	6.8, 7.6
Eating	2,870,635	3.7	3.4, 4.0
Transfer In-and-Out of Bed	5,501,113	7.0	6.6, 7.5
Use a Toilet	4,967,597	6.4	6.0, 6.8
2008 Wave			
Walk Across a Small Room	5,592,619	7.6	7.1, 8.0
Dressing	7,336,284	9.9	9.4, 10.4
Bathe	5,493,672	7.4	7.0, 7.9
Eating	2,613,724	3.5	3.2, 3.8
Transfer In-and-Out of Bed	4,785,320	6.5	6.0, 6.9
Use a Toilet	4,366,736	5.9	5.5, 6.3
2010 Wave			
Walk Across a Small Room	6,599,985	6.9	6.5, 7.3
Dressing	9,747,259	10.2	9.7, 10.7
Bathe	6,768,974	7.1	6.7, 7.5
Eating	3,393,671	3.6	3.3, 3.8
Transfer In-and-Out of Bed	6,342,415	6.7	6.2, 7.1
Use a Toilet	5,697,595	6.0	5.6, 6.4
2012 Wave			
Walk Across a Small Room	6,187,300	6.7	6.3, 7.1
Dressing	8,612,716	9.4	8.9, 9.9
Bathe	6,711,410	7.3	6.9, 7.7
Eating	3,263,672	3.6	3.3, 3.9
Transfer In-and-Out of Bed	6,076,062	6.6	6.2, 7.0
Use a Toilet	5,231,482	5.7	5.3, 6.1
2014 Wave			
Walk Across a Small Room	6,190,929	7.1	6.6, 7.5
Dressing	8,963,844	10.2	9.7, 10.8
Bathe	6,479,045	7.4	7.0, 7.8
Eating	3,361,605	3.8	3.5, 4.2
Transfer In-and-Out of Bed	5,718,824	6.5	6.1, 7.0
Use a Toilet	5,331,681	6.1	5.7, 6.5
2016 Wave			
Walk Across a Small Room	7,173,058	6.6	6.1, 7.0
Dressing	10,609,679	9.7	9.2, 10.2
Bathe	7,267,658	6.6	6.2, 7.1
Eating	3,613,224	3.3	3.0, 3.6
Transfer In-and-Out of Bed	6,739,294	6.2	5.8, 6.6
Use a Toilet	5,643,761	5.2	4.8, 5.5
2018 Wave			
Walk Across a Small Room	7,448,618	7.1	6.6, 7.6
Dressing	9,805,120	9.4	8.8, 9.9
Bathe	7,239,780	6.9	6.4, 7.4
Eating	3,589,205	3.4	3.1, 3.8
Transfer In-and-Out of Bed	7,216,450	6.9	6.4, 7.4
Use a Toilet	5,640,388	5.4	5.0, 5.8

**Table 3:** Overall prevalence estimates of individual basic self-care limitations.

Variables	Weighted Frequency (N)	Weighted Prevalence (%)	95% Confidence Interval
2006 Wave	13,974,905	17.8	17.2, 18.5
2008 Wave	12,946,466	17.4	16.8, 18.1
2010 Wave	16,366,128	17.1	16.5, 17.7
2012 Wave	15,077,479	16.4	15.8, 17.0
2014 Wave	15,214,239	17.3	16.7, 18.0
2016 Wave	17,553,261	16.0	15.4, 16.7
2018 Wave	17,314,873	16.5	15.8, 17.2

**Table 4:** Estimated prevalence of basic self-care disability by age group.

Variables	Weighted Frequency (N)	Weighted Prevalence (%)	95% Confidence Interval
Middle-Aged Adults			
2006 Wave	5,149,354	12.5	11.6, 13.5
2008 Wave	4,082,145	11.5	10.6, 12.5
2010 Wave	7,056,005	13.0	12.1, 13.8
2012 Wave	5,757,825	11.9	11.0, 12.8
2014 Wave	5,247,019	12.6	11.6, 13.6
2016 Wave	7,455,259	12.4	11.5, 13.3
2018 Wave	6,606,898	12.7	11.7, 13.8
Older Adults			
2006 Wave	8,825,551	23.7	22.8, 24.5
2008 Wave	8,864,321	22.8	21.9, 23.7
2010 Wave	9,310,123	22.6	21.7, 23.5
2012 Wave	9,319,654	21.3	20.5, 22.2
2014 Wave	9,967,220	21.6	20.7, 22.5
2016 Wave	10,098,002	20.4	19.4, 21.3
2018 Wave	10,707,975	20.2	19.2, 21.2

**Table 5:** Estimated prevalence of basic self-care disability by sex.

Variables	Weighted Frequency (N)	Weighted Prevalence (%)	95% Confidence Interval
Females			
2006 Wave	8,815,354	20.6	19.7, 21.4
2008 Wave	8,087,936	19.9	19.0, 20.8
2010 Wave	9,766,090	18.8	18.0, 19.7
2012 Wave	9,066,297	18.2	17.3, 19.0
2014 Wave	9,171,478	19.2	18.3, 20.1
2016 Wave	10,380,680	17.7	16.8, 18.6
2018 Wave	10,304,947	18.3	17.3, 19.3
Males			
2006 Wave	5,159,551	14.5	13.6, 15.5
2008 Wave	4,858,530	14.5	13.5, 15.5
2010 Wave	6,600,038	15.1	14.2, 16.0
2012 Wave	6,011,182	14.3	13.4, 15.2
2014 Wave	6,042,761	15.1	14.1, 16.0
2016 Wave	7,172,581	14.1	13.1, 15.0
2018 Wave	7,009,926	14.4	13.4, 15.5

**Table 6:** Estimated prevalence of basic self-care disability by race and ethnicity.

Variables	Weighted Frequency (N)	Weighted Prevalence (%)	95% Confidence Interval
Hispanic			
2006 Wave	1,297,182	22.2	19.8, 24.6
2008 Wave	1,296,863	22.8	20.4, 25.2
2010 Wave	1,963,894	25.1	22.7, 27.5
2012 Wave	1,873,650	23.9	21.5, 26.3
2014 Wave	1,907,366	24.8	22.3, 27.3
2016 Wave	2,420,158	22.0	19.8, 24.2
2018 Wave	2,505,630	23.2	20.8, 25.7
Non-Hispanic Black			
2006 Wave	1,978,255	27.3	25.2, 29.5
2008 Wave	1,581,411	23.0	21.0, 25.0
2010 Wave	2,442,499	25.3	23.5, 27.2
2012 Wave	2,250,392	24.5	22.5, 26.4
2014 Wave	2,233,141	25.3	23.2, 27.3
2016 Wave	2,714,225	23.6	21.7, 25.4
2018 Wave	2,695,928	24.4	22.4, 26.5
Non-Hispanic Other			
2006 Wave	385,993	18.3	13.9, 22.7
2008 Wave	381,606	19.4	14.6, 24.2
2010 Wave	643,229	19.9	15.9, 24.0
2012 Wave	564,850	17.6	13.7, 21.5
2014 Wave	594,089	19.1	15.1, 23.2
2016 Wave	895,401	15.5	12.6, 18.5
2018 Wave	988,893	18.1	14.4, 21.7
Non-Hispanic White			
2006 Wave	10,313,475	16.3	15.6, 17.0
2008 Wave	9,686,586	16.2	15.5, 17.0
2010 Wave	11,316,506	15.1	14.4, 15.8
2012 Wave	10,388,587	14.5	13.8, 15.2
2014 Wave	10,479,643	15.4	14.6, 16.1
2016 Wave	11,523,477	14.2	13.4, 14.9
2018 Wave	11,124,422	14.3	13.5, 15.2

## Data Availability

Data utilized in this investigation are publicly available from the Health and Retirement Study website.

## Supplementary Materials

The following supporting information can be downloaded at: https://www.mdpi.com/article/10.3390/epidemiologia4040040/s1, Supplementary Table S1: Results of trends analyses for basic activities of daily living.
