# Supplementary material for: Prevalence and Trends of Basic Activities of Daily Living Limitations in Middle-Aged and Older Adults in the United States
Source: Epidemiologia (Basel). 2023 Nov 9;4(4):483–91. doi: 10.3390/epidemiologia4040040 (PMC10660458; doi:10.3390/epidemiologia4040040)
Supplement: Supplementary file 1 [file epidemiologia-04-00040-s001.zip › epidemiologia-2576088-supplementary.pdf]

Supplementary Table S1. Results of trends analyses for basic activities of daily living.

Variables	Estimate	95% Confidence Interval	p-value
Overall Model			
Intercept	–1.40	–1.40, -1.30	<0.001
Wave	–0.01	–0.01, 0.01	0.52
Age Model			
Intercept	–2.20	–2.30, –2.10	<0.001
Wave	0.04	0.03, 0.05	<0.001
Older	1.06	0.90, 1.23	<0.001
Wave*Older	–0.04	-0.06, -0.03	<0.001
Gender Model			
Intercept	–1.20	-1.3, -1.1	<0.001
Wave	–0.01	-0.02, 0.01	0.11
Male	–0.46	-0.62, -0.30	<0.001
Wave*Male	0.01	-0.01, 0.03	0.06
Race and Ethnicity Model			
Intercept	–1.40	-1.50, -1.30	<0.001
Wave	–0.01	-0.02, 0.01	0.07
Hispanic	0.55	0.30, 0.81	<0.001
Non-Hispanic Black	0.62	0.40, 0.84	<0.001
Non-Hispanic Other	–0.05	-0.52, 0.42	0.82
Wave*Hispanic	–0.01	-0.04, 0.01	0.20
Wave*Non-Hispanic Black	–0.02	-0.04, 0.01	0.06
Wave*Non-Hispanic Other	0.01	-0.03, 0.05	0.62
